# Bioactivity of Common Pesticidal Plants on Fall Armyworm Larvae (*Spodoptera frugiperda*)

**DOI:** 10.3390/plants9010112

**Published:** 2020-01-15

**Authors:** Kelita Phambala, Yolice Tembo, Trust Kasambala, Vernon H. Kabambe, Philip C. Stevenson, Steven R. Belmain

**Affiliations:** 1 Department of Crop and Soil Sciences, Lilongwe University of Agriculture and Natural Resources, Lilongwe Box 219, Malawi; kelitaphambala@yahoo.com (K.P.); ytembo@bunda.luanar.mw (Y.T.); tdonga@luanar.ac.mw (T.K.); kabambev@gmail.com (V.H.K.); 2 Natural Resources Institute, University of Greenwich, Central Avenue, Chatham Maritime, Kent ME4 4TB, UK; p.c.stevenson@gre.ac.uk; 3 Biological Chemistry and In Vitro Research, Royal Botanic Gardens, Richmond TW9 3AB, UK

**Keywords:** botanical pesticide, pesticidal plant, pest management, invasive species, agro-ecological intensification, sustainable agriculture

## Abstract

The fall armyworm (FAW), *Spodoptera frugiperda* (Lepidoptera: Noctuidae) is a recent invasive pest species that has successfully established across sub-Saharan Africa where it continues to disrupt agriculture, particularly smallholder cereal production. Management of FAW in its native range in the Americas has led to the development of resistance to many commercial pesticides before its arrival in Africa. Pesticide use may therefore be ineffective for FAW control in Africa, so new and more sustainable approaches to pest management are required that can help reduce the impact of this exotic pest. Pesticidal plants provide an effective and established approach to pest management in African smallholder farming and recent research has shown that their use can be cost-beneficial and sustainable. In order to optimize the use of botanical extracts for FAW control, we initially screened ten commonly used plant species. In laboratory trials, contact toxicity and feeding bioassays showed differential effects. Some plant species had little to no effect when compared to untreated controls; thus, only the five most promising plant species were selected for more detailed study. In contact toxicity tests, the highest larval mortality was obtained from *Nicotiana tabacum* (66%) and *Lippia javanica* (66%). Similarly, in a feeding bioassay *L. javanica* (62%) and *N. tabacum* (60%) exhibited high larval mortality at the highest concentration evaluated (10% *w*/*v*). Feeding deterrence was evaluated using glass-fibre discs treated with plant extracts, which showed that *Cymbopogon citratus* (36%) and *Azadirachta indica* (20%) were the most potent feeding deterrents among the pesticidal plants evaluated. In a screenhouse experiment where living maize plants infested with fall armyworm larvae were treated with plant extracts, *N. tabacum* and *L. javanica* were the most potent species at reducing foliar damage compared to the untreated control whilst the synthetic pesticide chlorpyrifos was the most effective in reducing fall armyworm foliar damage. Further field trial evaluation is recommended, particularly involving smallholder maize fields to assess effectiveness across a range of contexts.

## 1. Introduction

The fall armyworm, *Spodoptera frugiperda* (J.E. Smith) (Lepidoptera: Noctuidae) (FAW) is a polyphagous pest that is invasive and now widely established across sub-Saharan Africa. Although similar to the native African armyworm, *Spodoptera exempta* (Walker), FAW is more likely to persist year-round once established, attacking a much wider range of cereals as well as more than 100 other horticultural crops [1]. Native to North and South America, FAW was first reported in West and Central Africa in 2016 [2] and is now reported in at least 44 African countries [3]. FAW is a heavy foliage feeder that can cause 100% loss of cereal crops [4]. In the absence of effective control methods, potential maize yield losses caused by FAW have been estimated between 8.3 and 20.6 million metric tons per year in just 12 maize-producing countries in Africa. This represents a range of 21–53% of annual maize production averaged over a three-year period. The value of these losses was estimated at between US $2481 million and US $6187 million [5].

Current armyworm control relies on the use of synthetic pesticides; however, widespread over-use and misuse in the Americas have resulted in considerable problems with insecticide resistance particularly among the carbamates, pyrethroids and organophosphates [6] on which many African farmers rely. As African farmers have a long history of using plants with pesticidal properties [7,8,9,10,11], options for developing botanical biopesticides for FAW control may be more realistic than in other regions [12]. Recent research has evaluated several abundant pesticidal plant species, confirming that their use in smallholder farming can result in comparable yield to that when using commercial synthetics, without the severe environmental damage often associated with synthetic compounds [13,14,15]. With a need to develop new, effective and agro-ecologically sustainable methods for controlling FAW in Africa, we set out to screen some of the more promising pesticidal plant species where considerable knowledge already exists on their abundance, phytochemistry and safe use. The specific objective of the research presented here was to evaluate potential effects of pesticidal plants on the larval stage, assessing direct toxicity as well as post-ingestive toxicity and feeding deterrence. Finally, the most promising pesticidal plant extracts were evaluated in controlled trials using FAW-infested maize plants to determine whether the plant extracts reduced foliar damage under cropping conditions.

## 2. Results and Discussion

### 2.1. Contact Toxicity and Feeding Bioassays with Ten Pesticidal Plant Species

**Figure 1:**
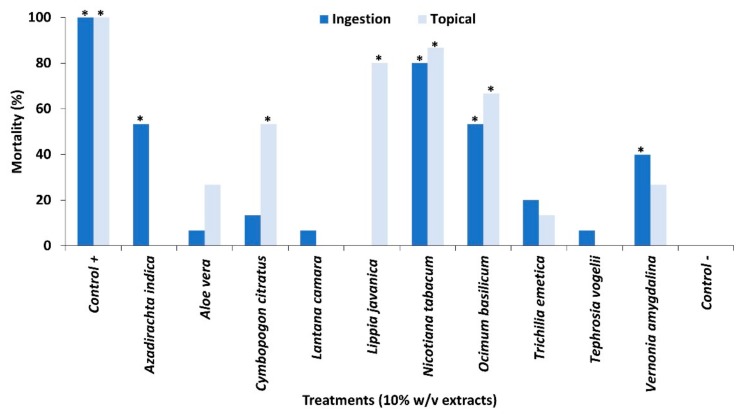
Mortality of 2nd instars when exposed to extracts of pesticidal plant either topically applied through a contact toxicity bioassay or through ingestion in a feeding bioassay. Treatments different from the untreated control (*p* < 0.05) are indicated by *. Significant differences are presented in Table S1.

Water extracts (10% *w*/*v*) of ten common pesticidal plants which are regularly used by smallholder farmers showed variable effects on larval mortality (Figure 1). *Tephrosia vogelii* Hook.f. and *Lantana camara* L. showed very low mortality (<10%) in both feeding and contact toxicity bioassays which was surprising since previous research on both of these plant species demonstrated high and consistent efficacy against a range of pest species using the same extraction methods and plant sources and the same biologically active phytochemicals [13,14,15,16,17]. Low mortality (<40%) was also observed with *Vernonia amygdalina* Delile followed by *Aloe vera* (L.) Burm.f. and *Trichilia emetica* Vahl, despite evidence of efficacy against other target insect pests [11,18,19,20,21]. The most effective plant species were *Azadirachta indica* A. Juss., *Cymbopogon citratus* (DC.) Stapf, *Lippia javanica* (Burm.f.) Spreng., *Nicotiana tabacum* L. and *Ocimum basilicum* L. which caused at least 50% mortality through at least one bioassay [22]. Although some plant species had an effect through one application method only (*A. indica* and *L. javanica*), overall, the application method led to comparable effects for most plant species, which is verified through statistical analyses (Table S1). Other research on the evaluation of botanicals against FAW in Ethiopia [1], showed *N. tabacum* to cause 50% mortality to 3rd instars after 72 h exposure, which is considerably lower than the mortality observed in our bioassay. Further, this work found that *A. indica* and six other plant species were more effective than *N. tabacum* with mortality rates of 75–98%.

### 2.2. Contact Toxicity and Feeding Bioassays with Five Pesticidal Plant Species

**Figure 2:**
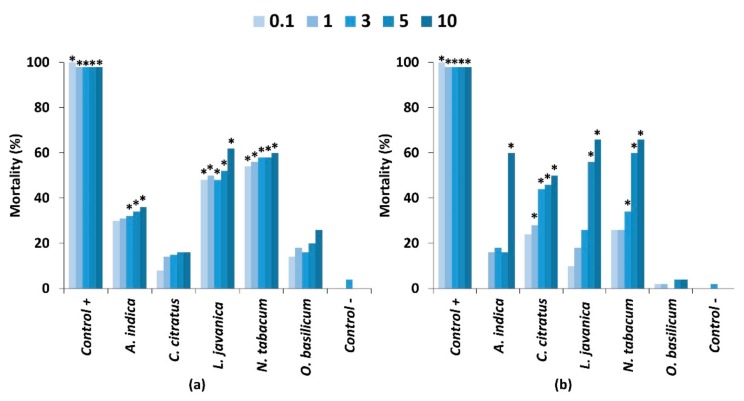
Mortality of fall armyworm 2nd instars seven days after exposure to five different concentrations of pesticidal plant extracts when applied through (**a**) a feeding bioassay and (**b**) a contact toxicity bioassay. Treatments differing significantly from the untreated control (*p* < 0.05) are indicated by *. Significant differences are presented in Table S1.

Plant extracts applied to glass fibre discs showed that the five most active plant species from the previous bioassay caused significantly greater mortality in comparison to the untreated control (Figure 2a). At the highest concentration (10%), *L. javanica* (62%) and *N. tabacum* (60%) exhibited high larval mortality. The lowest mortality was observed from *C. citratus* (16%) and *O. basilicum* (26%). Although there was a slight dose dependent effect, this was not significant for any of the plant extracts (*p* < 0.05; Table S1). Some differences in efficacy were observed in comparison to the first trial where extracts were prepared in water. Mortality trends between water and methanol extracts were similar with the exception of *L. javanica* where no mortality was observed in the feeding bioassay when applying the water extracts to discs. The reasons for this difference are not clear but may be caused by differences in methodology, in which water extract was presented on maize leaves to reflect farmer practices, whereas the methanol extract was applied to glass fibre discs to more easily assess potential effects of extract concentration. However, the differences are more likely due to the extraction efficiency of the different solvents where hydrophobic compounds with bioactivity were more efficiently extracted by methanol than water [11,13,16,23]. The lack of a clear dose effect as well as differences in mortality between the trials using water or methanol could also be caused by differences in larval feeding rates through feeding deterrence behaviours.

The topical application of plant extracts to FAW larvae showed a strong dose response for four out of the five plant species (Figure 2b; Table S1), whereas mortality from *O. basilicum* (4%) was not significantly different from the untreated control (*p* < 0.5). As expected, the 10% concentration exhibited high larval mortality of 50–66% for *N. tabacum* (66%), *L. javanica* (66%), *A. indica* (60%) and *C. citratus* (50%). However, the positive control of chlorpyrifos was superior to all plant extracts, causing nearly 100% mortality. Although these extracts were made using methanol, the mortality rates observed were not significantly different from the application of water extracts in the first trial (*p* < 0.05).

**Figure 3:**
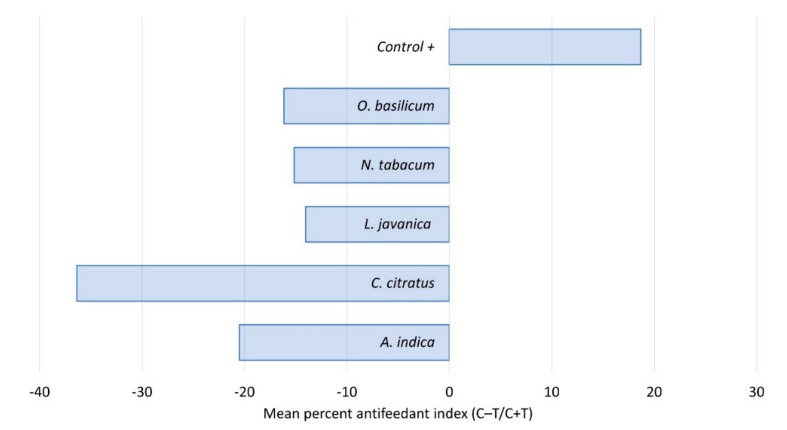
Antifeedant activity of five plant species extracts fed to fall armyworm 2nd instars, showing percent of feeding deterrence after 48 h. C = control; T = treated.

All of the five plant extracts used in this trial showed some degree of deterrence (Figure 3). The most potent feeding deterrents were *C. citratus* and *A. indica*. Although mortality appears to be relatively low with these two species, feeding deterrent compounds could help to reduce crop damage. In agreement with our observations, other studies demonstrated the deterrent effects of several *Cymbogon* species as well as *A. indica*. In a binary choice test, the essential oils isolated from *C. nardus*, *C. flexuosus* and *C. martini* exhibited strong antifeedant activity against *Acharia fusca* and *Euprosterna elaeasa* [24]. The antifeedant properties of *A. indica* are well established, particularly for a range of lepidopteran pests [25,26,27].

**Figure 4:**
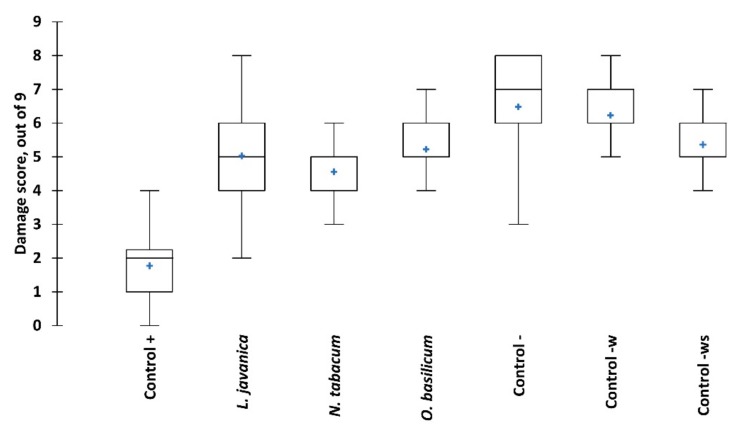
Fall armyworm damage to maize plants when exposed to different treatments over eight weeks. Boxes represent mean and 95% confidence intervals, tails are max. and min. values, blue crosses are median values. Significant differences are presented in Table S1.

Trials evaluating the three most promising pesticidal plant species for their ability to control FAW larvae on living maize plants showed significant differences in effect among the treatments (Figure 4). High foliar damage was observed in the negative controls (untreated, water and water plus 0.1% soap) with mean leaf damage scores of 6.5, 6.3 and 5.4, respectively. The lowest foliar damage score was observed in *N. tabacum* treatment (4.6); however, the slightly higher scores for *L. javanica* (5) and *O. basilicum* (5.2) were not significantly different from *N. tabacum*. The synthetic pesticide, chlorpyrifos, was the most effective in reducing FAW foliar damage with a mean score of 1.8.

The observed reduction in foliar damage may be attributed to a combination of toxicity, repellent and antifeedant effects of the plant extracts, with similar results observed from other studies [1]. The plant extracts did not reduce FAW damage as much as the synthetic pesticide chlorpyrifos, but most other studies on the use of pesticidal plants show similar lower mortality and damage rates when using natural pesticides in comparison to synthetic pesticides [13,15]. As most crops can compensate for some limited pest damage, further studies are required to determine whether these pesticidal plant treatments are able to maintain yield at comparable levels to synthetic pesticide use, which has been reported for a number of legume crops [14], cabbages [28,29] and sorghum [30].

## 3. Materials and Methods

### 3.1. Fall Armyworm Rearing

FAW larvae were collected from maize fields around Mitundu, Lilongwe District, Malawi (latitude 14°11′ S longitude 33°46′ E, elevation of 1100 metres above sea level (m a.s.l.). To establish a large colony the larvae were initially reared on portions of young maize leaves; however, once established, larvae were reared on an established artificial diet. The diet was composed of maize leaf powder, common bean powder, brewer’s yeast, ascorbic acid, sorbic acid, methyl-*p*-hydroxybenzoate, vitamin E capsules, sucrose, formaldehyde and agar [31].

Neonates and 2nd instars were reared in 500 mL plastic containers containing young maize leaves, which were renewed daily. At the 2nd instar, the larvae were transferred to individual plastic containers (100 mL) to reduce cannibalism until pre-pupal stage and fed on an artificial diet which was changed every week until pupation. The pupae were harvested and transferred in Petri dishes lined with tissue paper and placed in adult emergence cages (mosquito netting around an 18 × 18 × 18 cm frame). After adult emergence, the moths were fed on honey from a honey-soaked wad of cotton wool in a container placed in each cage. Eggs laid on filter paper in the cages were removed daily and were disinfected by dipping them in 10% formaldehyde for 15 min. The eggs were then rinsed thoroughly with distilled water and dried on filter paper. Thereafter, eggs were placed in small containers until they hatched to repeat the rearing process [31].

### 3.2. Plant Material Collection and Extract Preparation

Initial plant screening was carried out with ten pesticidal plant species: *A. indica*, *O. basilicum*, *N. tabacum*, *C. citratus*, *T. vogelii*, *A. vera*, *L. camara*, *T. emetica*, *V. amygdalina* and *L. javanica*. Considerable phytochemical and efficacy knowledge was recorded by our group and others on all the material used of these species [10,15,17,18,20,24,32,33,34,35,36,37] which were sourced from the same locations with samples from four locations around Mitundu, Lilongwe District, Malawi combined to control for potential chemical variation across space [37,38]. The leaves of all plant species were collected from the wild from these known locations. Plant materials were shade dried, ground to a fine powder and kept in cool dark conditions until required. To produce 10% *w*/*v* extracts, 100 g of each plant powder were extracted in 1 L of water containing 0.1% soap for 24 h at room temperature. Thereafter the extracts were filtered and used immediately in bioassays. This trial included two control treatments, a positive control of chlorpyrifos and a negative control of water plus 0.1% soap.

Based on these bioassay results, five of the ten plant species were selected for further research: *A. indica*, *O. basilicum*, *N. tabacum*, *C. citratus* and *L. javanica*. In order to improve the extraction efficiency, the extracts were prepared by weighing 300 g of plant powder into 1.5 L of methanol for 24 h. Extracts were then filtered and placed in a fume hood for 24 h to allow the methanol to evaporate, leaving behind the extracted residue. Dried residue was weighed on an analytical scale and then resolubilized in acetone to make five concentrations of 10%, 5%, 3%, 1% and 0.1% *w*/*v* [39]. This trial included two control treatments, a positive control of chlorpyrifos and a negative control of acetone only.

The evaluation of FAW survival on living maize plants was carried out using the three best plant species: *O. basilicum*, *N. tabacum* and *L. javanica*. Aqueous extracts were prepared at 10% *w*/*v* using the extraction process described above in the first screening experiment. Four control treatments were also used in this study: chlorpyrifos as a positive control and three negative controls of no treatment application, water only and water with 0.1% soap.

### 3.3. Bioassay Methods

Contact toxicity was performed by means of topical application where 10 μL of the extract were applied topically on the bodies of the larvae using a 20 μL pipette. The larvae were then individually placed in plastic bottle tops containing plain artificial diet and covered with foil paper. Chlorpyrifos 48 EC was used as positive control using the manufacturer recommended rate of 20 mL of chlorpyrifos in 40 L of water while acetone or water was used as the negative control. Each replicate contained ten 2nd instars, with five replicates per each treatment and concentration evaluated. Final mortality data were recorded seven days afterwards by counting the number of dead larvae, with mortality data corrected using Abbott’s formula [40].

The initial feeding bioassay screening ten plant species was carried out by dipping portions of young maize leaves into each extract, waiting one hour for the extract to dry and then placing five 2nd instars on the treated leaves to feed, three replicates per treatment. Treated maize leaves were replaced daily for seven days, with mortality data collected and corrected with Abbott’s formula.

The subsequent feeding bioassay screening the shortlisted five plant species was carried out using previously reported methods [41]. Aliquots (100 μL) of 0.05 M sucrose in acetone were applied to individual glass-fibre discs (Whatman 2.1 cm diameter) and left to dry before aliquots (100 μL) of the plant extracts in acetone were applied to each disc. Once dry, the discs were weighed with an analytical balance, placed in individual containers and one 2nd instar was introduced with 10 larvae per treatment. Two control treatments were used: sucrose only and chlorpyrifos. After 48 h the remainder of the disc not eaten by the larvae was weighed and any living larvae were transferred to plain diet in individual containers. Mortality data were collected seven days from the start of the trial with mortality data corrected using Abbott’s formula. A feeding deterrence index was calculated from the weights of control (C) and treated (T) discs using the following formula: Feeding deterrence = (C − T)/(C + T) × 100 [42].

The final experiment evaluating FAW damage to living maize plants was carried out using maize variety sc403 planted in pots maintained in a screenhouse. Five seeds were planted per pot and were later thinned to three. Basal dressing fertiliser of 23:21:0 + 4 s was applied at seven days after planting at a rate of 100 kg/ha while a topdressing fertilizer of urea was applied at four weeks after planting at a rate of 159 kg/ha. All agronomic practices including watering and hand weeding were consistent across all maize plant pots. Maize plants were infested with 2nd instars at twenty days after seedling emergence. Each plant was infested with five larvae and the larvae were spaced at different leaf nodes to avoid cannibalism. Artificial infestation was done early in the morning to avoid exposing the larvae to harsh environments [31]. After infestation, plant pots were caged individually in cages of size 1.8 m × 0.6 m × 0.6 m to prevent the movement of larvae from one treatment to another. The experiment was laid out in a randomized complete block design replicated ten times where the cages acted as blocks. Hand-held plastic sprayer bottles were used to apply the treatments, ensuring consistent coverage of each plant. Treatments were first applied 48 h after infestation to allow the larvae to settle down and establish [43]. Subsequent applications were carried out at seven-day intervals. Foliar damage data were collected at an interval of seven days beginning from the first day after spraying. Using published methods [44], FAW foliar damage severity was recorded on an individual plant basis using a scale of 0–9 where 0 means no visible leaf damage, 1 = only pin-hole damage to the leaves, 2 = pin-hole and shot-hole damage to leaves, 3 = small elongated lesions (5–10 mm) on 1–3 leaves, 4 = midsized lesions (10–30 mm) on 4–7 leaves, 5 = large elongated lesions (>30 mm) or small portions eaten on 3–5 leaves, 6 = elongated lesions (>30 mm) and large portions eaten on 3–5 leaves, 7 = elongated lesions (>30 cm) and 50% of leaf eaten, 8 = elongated lesions (30 cm) and large portions eaten on 70% of leaves and 9 = most leaves have long lesions.

### 3.4. Data Analysis

Experiments were carried out following completely randomised block designs. Effects of treatments and their interactions observed were subjected to two-way analysis of variance. The means of treatments and interactions were compared using Tukey’s honest significant difference (HSD) test at the 95% confidence interval. All the analyses were done using XLSTAT version 2019.2.2.59614 (Addinsoft, 2019); XLSTAT statistical and data analysis solution (Boston, MA, USA). Statistical outputs are provided in Table S1.

## 4. Conclusions

Recommendations from this research suggest that some relatively safe pesticidal plant species could provide an agro-ecologically sustainable pest management option for the exotic invasive FAW in Africa. Out of the original ten candidate plant species evaluated, four of these merit further investigation: *Azadirachta indica*, *Ocimum basilicum*, *Cymbopogon citratus* and *Lippia javanica*. These four plant species are cosmopolitan and frequently cultivated, so sustainable supplies for large-scale production would be feasible. Although *Tephrosia vogelii* did not show significant efficacy in our trials, further research should be recommended to confirm these results as *T. vogelii* is being recommended for fall armyworm control due to is known efficacy against a range of pests. Considerable knowledge on the chemistry of these plants is reported. Furthermore, *O. basilicum*, *C. citratus* and *L. javanica* are consumed as spices and teas, whilst *Azadirachta indica* has well-established low mammalian toxicity. Despite considerable work on its biopesticidal effects, *Nicotiana tabacum* is arguably the plant species with the highest vertebrate toxicity, well-known for the effects of nicotine and related alkaloids. However, despite potential human toxicity dangers, *N. tabacum* is being pursued as one of several potential botanical options for FAW control in several African countries, and thus merits further investigation regarding its safe use and non-target effects. Evidence from our work and previous research repeatedly shows that pesticidal plants do not cause mortality rates comparable to synthetic pesticides. However, the trade-off between lower mortality for lower environmental persistence needs to be seriously considered, particularly as there is growing evidence that less toxic natural pesticides can help facilitate natural pest regulation whilst not significantly sacrificing crop yield. The next step in evaluating the use of pesticidal plants for FAW control in Africa will require systematic trials under farmer field conditions that can assess their cost-benefits and impact on crop damage and yield in comparison to commercial synthetic pesticides.

## Supplementary Materials

The following are available online at https://www.mdpi.com/2223-7747/9/1/112/s1, Table S1: Statistical analyses of fall armyworm bioassays evaluating pesticidal plants using two-way analysis of variance followed by Tukey’s HSD test to separate the means. Means followed by the same letter in an experiment are not significantly different from each other.
