# Supplementary material for: Bioactivity of Common Pesticidal Plants on Fall Armyworm Larvae (Spodoptera frugiperda)
Source: Plants (Basel). 2020 Jan 15;9(1):112. doi: 10.3390/plants9010112 (PMC7020173; doi:10.3390/plants9010112)
Supplement: Supplementary file 1 [file plants-09-00112-s001.pdf]

Table S1. Effects of treatments and their interactions subjected to 2-way Analysis of Variance. The means of treatments and interactions were compared using the Tukey HSD test at the 95% confidence interval. In each experiment, mean values followed with the same letter are not different from each other.

* indicates significance at P < 0.05; ** indicates significance at P < 0.0001.

Water extracts	Contact toxicity, percent mortality 10%	Feeding toxicity, percent mortality 10%
Control +	100 a	100 a
A. indica	0 d	53 ab
A. vera	26 bcd	6 c
C. citratus	53 abc	13 c
L. camara	0 d	6 c
L. javanica	80 a	0 c
N. tabacum	86 a	80 ab
O. basilicum	66 ab	53 ab
T. emetica	13 cd	20 c
T. vogelii	0 d	6 c
V. amygdalina	26 bcd	40 bc
Control -	0 d	0 c
F treatment	14.24**	9.19**
F application method	0.47	
F treat. * app. method	11.06**	

	Contact toxicity, percent mortality					Feeding toxicity, percent mortality				
Methanol extracts	0.1%	1%	3%	5%	10%	0.1%	1%	3%	5%	10%
Control +	100 a	98 a	98 a	98 a	98 a	100 a	98 a	98.000 a	98 a	98 a
A. indica	0 h	16 fgh	18 efgh	16 fgh	60 bc	30 bcdefghi	31 bcdefgh	32 defgh	34 bcdefgh	36 bcdefgh
C. citratus	24 defgh	28 defg	44 bcde	46 bcd	50 bcd	8 hi	14 hi	15 hi	16 hi	16 hi
L. javanica	10 fgh	18 efgh	26 defgh	56 bc	66 b	48 bcdefg	50 bcdef	48 bcdefg	52 bcde	62 b
N. tabacum	26 defgh	26 defgh	34 cdef	60 bc	66 b	54 bcd	56 bcd	58 bc	58 bc	60 b
O. basilicum	2 gh	2 gh	0 h	4 gh	4 gh	14 hi	18 fghi	16 ghi	20 efghi	26 cdefghi
Control -	0 h	0 h	2 h	0 h	0 h	0 i	0 i	4 i	0 i	0 i
F treatment	92.05**					168.71**				
F concentration	3.31*					0.18				
F treat. * conc.	46.461**					27.07**				

Water extracts 10% w/v) on infested maize plants	Leaf damage index
Control +	1.77 d
L. javanica	5.02 bc
N. tabacum	4.56 c
O. basilicum	5.22 bc
Control -	6.48 a
Control - w	6.22 a
Control - ws	5.36 b
F treatment	90.89**
